# Reversible hypogonadotropic hypogonadism in men with the fertile eunuch/Pasqualini syndrome: A single-center natural history study

**DOI:** 10.3389/fendo.2022.1054447

**Published:** 2022-11-02

**Authors:** Andrew A. Dwyer, Maria Stamou, Isabella R. McDonald, Ella Anghel, Kimberly H. Cox, Kathryn B. Salnikov, Lacey Plummer, Stephanie B. Seminara, Ravikumar Balasubramanian

**Affiliations:** ^1^ Boston College William F. Connell School of Nursing, Chestnut Hill, MA, United States; ^2^ Massachusetts General Hospital – Harvard Center for Reproductive Medicine, Massachusetts General Hospital, Boston, MA, United States; ^3^ Reproductive Endocrine Unit, Massachusetts General Hospital, Boston, MA, United States; ^4^ Boston College Lynch School of Education and Human Development, Department of Measurement, Evaluation, Statistics and Assessment, Chestnut Hill, MA, United States

**Keywords:** genetics, reversible hypogonadism, puberty, male infertility, Kallmann syndrome, hypogonadotropic hypogonadism, reproduction

## Abstract

Congenital hypogonadotropic hypogonadism (HH) is a heterogeneous genetic disorder characterized by disrupted puberty and infertility. In most cases, HH is abiding yet 10-15% undergo reversal. Men with HH and absent and partial puberty (i.e., testicular volume <4mL and >4mL respectively) have been well-studied, but the rare fertile eunuch (FE) variant remains poorly characterized. This natural history study of 240 men with HH delineates the clinical presentation, neuroendocrine profile, rate of reversal and genetics of the FE variant. We compared three HH groups: FE (n=38), absent puberty (n=139), and partial puberty (n=63). The FE group had no history of micropenis and 2/38 (5%) had cryptorchidism (*p*<0.0001 vs. other groups). The FE group exhibited higher rates of detectable gonadotropins, higher mean LH/FSH levels, and higher serum inhibin B levels (all *p*<0.0001). Neuroendocrine profiling showed pulsatile LH secretion in 30/38 (79%) of FE men (*p*<0.0001) and 16/36 (44%) FE men underwent spontaneous reversal of HH (*p*<0.001). The FE group was enriched for protein-truncating variants (PTVs) in *GNRHR* and *FGFR1* and 4/30 (13%) exhibited oligogenic PTVs. Findings suggest men with the FE variant exhibit the mildest neuroendocrine defects of HH men and the FE sub-type represents the first identified phenotypic predictor for reversible HH.

## Introduction

Gonadotropin-releasing hormone (GnRH) is referred to as the ‘pilot light’ of reproduction for its role in initiating and maintaining activity of the hypothalamic-pituitary-gonadal (HPG) axis (1). The HPG axis is active during minipuberty (i.e., late fetal-early neonatal life), falls quiescent during childhood, and reactivates in puberty - culminating in sexual maturation and reproductive capacity. Specialized hypothalamic neurons secrete GnRH in a pulsatile manner triggering the release of gonadotropins (i.e., luteinizing hormone [LH] and follicle-stimulating hormone [FSH]) from the anterior pituitary that stimulate gonadal secretion of testosterone and spermatogenesis in males (1, 2). Isolated defects in GnRH secretion or action can disrupt HPG axis function and result in hypogonadotropic hypogonadism (HH). HH is more common in men and biochemically, HH in men is characterized by frankly low serum testosterone (T) in the setting of low or inappropriately low gonadotropin levels. Importantly, men with HH do not represent a singular, monolithic group and their clinical features are heterogeneous with variable pubertal phenotypes at presentation and both abiding and reversible HH during adulthood (1, 2).

Initial approaches to identify HH sub-types were based on non-reproductive clinical characteristics such as olfactory function to differentiate normosmic (nHH) from Kallmann syndrome (i.e., HH with anosmia) (3). However, the HH phenotype in men is equally heterogeneous with respect to their reproductive phenotypes. Reproductive phenotypes recognized in HH men include neonatal defects such as cryptorchidism (maldescended testes) and micropenis, and variable degrees of testicular development (testicular volume, TV) during adolescent puberty. Non-reproductive phenotypes also occur at variable rates and include skeletal/dental defects, unilateral renal agenesis, synkinesia (mirror movements), and sensory deficits (i.e., anosmia, hearing loss, eye movement disorders) (1, 2). Similar to the variable clinical presentation, HH is also genetically heterogeneous. To date, more than 60 loci have been identified for HH and subjects harboring either a single gene defect with Mendelian inheritance or complex inheritance patterns that include digenic/oligogenic forms (4). Unlike many rare disorders, treatments (i.e., T replacement) are available to effectively induce secondary sexual characteristics (i.e., virilization) and approximately 75-80% of men can achieve fertility with either exogenous gonadotropin therapy or pulsatile GnRH administered by a microinfusion pump (5). However, outcomes of fertility-inducing treatment can be variable (6). Men with a history of cryptorchidism and/or absent spontaneous pubertal development (i.e., TV <4mL) tend to have poorer fertility outcomes (3). Although HH is typically abiding and lasts throughout life, approximately 10-15% of men undergo a spontaneous reversal of HH with recovery of HPG axis function and fertility (7). Currently, the precise clinical or genetic predictors of reversal have yet to be elucidated. Since the degree of pubertal development is linked to adult fertility outcomes, it is possible that pubertal phenotypic variation may also determine or predict future reversal of HH. Thus, it can be hypothesized that men with the mildest pubertal phenotypic presentation are more likely to exhibit reversible HH later in life.

A sub-set of men with HH exhibit the so-called ‘fertile eunuch’ (FE) phenotype. Rodolfo Pasqualini first reported a case of ‘hypoandrogenism with spermatogenesis’ in 1950 coining it “Pasqualini syndrome” (8). The clinical constellation consisted of eunuchoidism (i.e., arm span greater than height), near-normal/normal TV with normal spermatogenesis on biopsy (i.e., mature spermatozoa, high proportion of seminiferous tubes, and undifferentiated/immature Leydig cells), therapeutic response to human chorionic gonadotropin (hCG), and normal urinary gonadotropin levels (9). In the ensuing 70 years, 32 publications have reported the FE variant either in case reports (n=19), case series (n=6), or within larger cohorts of men with HH (n=7) (**Supplemental Materials Table 1**). Cumulatively, only 61 FE cases have been reported to date in the literature. Notably, the definition of FE has evolved and the testicular biopsy parameters are no longer considered essential. Rather, FE can be clinically defined by frank hypogonadism (<100 ng/dL, <3.5 nmol/L), undervirilization, eunuchoidal proportions, near-normal TV, and normal serum FSH levels (**Supplemental Materials Table 1**).

Given the heterogeneous clinical presentation of HH, variable response to fertility-inducing treatment, and advances in our molecular understanding of HH we aimed to chart the natural history of the FE sub-phenotype. Specifically, we delineate the clinical presentation, neuroendocrine profile, genetics, and rate of reversal in the largest reported cohort of FE men. We posit that clearly identifying HH sub-sets and their phenotypic/genetic correlates will improve diagnostic precision, inform clinical decision-making, and identify more targeted approaches to care and counseling for HH.

## Materials and methods

This retrospective study was reviewed and approved by the institutional review board of the Massachusetts General Hospital (MGH). All subjects provided written informed consent prior to study participation.

### Subjects

The cohort was drawn from male subjects with HH studied at the Massachusetts General Hospital Reproductive Endocrine Unit (1980-2020). Subjects were considered to have HH based on failure to undergo spontaneous puberty by the age of 18 years, frankly hypogonadal serum sex steroid levels (<100 ng/dL [<3.5 mmol/L]) in the setting of low/inappropriately normal gonadotropins (LH/FSH), and no evidence of an underlying cause of hypogonadism (i.e., no functional cause, normal iron binding studies, otherwise normal anterior pituitary function, normal CT/MRI imaging of the hypothalamo-pituitary region) (3). To define the criteria for FE, we reviewed the existing literature (**Supplemental Materials Table 1**). Patients were classified as having the FE variant if they met the above criteria and exhibited detectable serum FSH levels and partial spontaneous puberty (as evidenced by TV≥8 mL by Prader orchidometer) without previous gonadotropin therapy (or pulsatile GnRH) and/or presence of sperm on seminal fluid analysis (if able to produce an ejaculate).

### Baseline clinical and biochemical assessment

A detailed health history and evidence of HH-associated reproductive and non-reproductive phenotypes were recorded for each subject. Subjects underwent quantitative olfactory testing using the University of Pennsylvania Smell Identification Test (UPSIT) (10). A score of at least the fifth centile based on sex and age was considered normosmic (i.e., nHH). Individuals scoring <5^th^ percentile were considered anosmic (i.e., Kallmann syndrome). Physical examination included assessment of body habitus (i.e., eunuchoidal proportions) and testicular volume by Prader orchidometer. A TV <4 mL was considered prepubertal - consistent with absent spontaneous puberty. Men with TV ≥4 mL were classified as partial spontaneous puberty. Following treatment-specific washout (i.e., 4-8 weeks), subjects underwent detailed neuroendocrine profiling with overnight blood sampling every 10 min for 12-24 hours to assess endogenous GnRH-induced LH secretion, as previously described (3). The LH pulses were determined using the modified Santen and Bardin methodology (11, 12). Serum testosterone, FSH, mean LH, and inhibin B were measured in study pools of equal aliquots of all collected samples during the sampling period.

### Hormone measurements

As these studies spanned an extended period of time, different immunoassay systems were used to measure the gonadotropins, as previously described (3, 13). Serum LH and FSH were measured by microparticle enzyme immunoassays using an AxSYM instrument (Abbott Laboratories, Chicago, IL, USA) with intra- and interassay coefficients of variation (CVs) <7.5%. The limit of detection was 1.6 IU/L based on an HMG standard, equivalent to 0.34 mIU/ml LH or 0.66 mIU/ml FSH based on pituitary standards. For consistency with previous reports, the Second International Reference Preparation was used as the HMG reference standard. Serum T concentrations were measured using the DPC Coat-A-Count RIA kit (Diagnostics Products Corporation, Los Angeles, CA, USA) with intra- and interassay CVs <10%. Inhibin B was measured using a double-antibody ELISA (Serotec, Oxford, UK), which has a level of detection of 15.6 pg/mL, an intraassay CV <6% and interassay CV <18%.

### Evaluation of HH reversal

Seminal fluid analyses were performed on men able to produce an ejaculate and analyzed according to WHO parameters (14). Sustained reversal of HH is defined as previously reported (15), i.e., normal, adult endogenous serum T levels off treatment (≥270 ng/dL,[>9.4 nmol/L]). Subjects were evaluated for spontaneous recovery of their HPG axis (i.e., serum hormone levels, seminal fluid analysis) following appropriate treatment-specific washout.

### Genetic sequencing and analysis

Peripheral blood samples were collected from participants to perform exome sequencing (ES, Broad Institute, Cambridge, MA, USA). We used GATK best practices (Broad Institute) (16) to align exome sequencing data to the reference genome (hg19), conduct initial quality control, and preform variant calling algorithms. Single nucleotide variant (SNV) calling and joint genotyping were performed using GATK components HaplotypeCaller and GenotypeGVCFs. Variant call format (VCF) files were annotated using SnpSift 4.3k and Ensembl VEP release 93. Rare SNVs were defined by a minor allele frequency (MAF) <0.1% in the control database - gnomAD (17). Copy number variants (CNVs) were called from ES with utilization of the GATK-gCNV pipeline as previously described (18).

### Definition of severity of genetic defects in IHH genes

We included both single nucleotide variants (SNVs)/indels and copy number variants (CNVs) in our genetic analyses. Genetic severity was defined for each of the 62 IHH-implicated genes (**Supplemental Material Table 2**) based on their reported mode of inheritance, underlying variant type, and minor allele frequency (MAF) (17). For autosomal genes, SNVs/indels with MAF of <0.1% and CNVs with MAF <1% in gnomAD database were included. For X-linked recessive genes, hemizygous variants with MAF <1% were included in the analysis. Similarly, for autosomal recessive genes, homozygous/compound heterozygous variants (MAF <1%) were considered causative for the analysis. For monogenic variants in those genes, we focused our analysis on “severe genetic defects”, i.e. protein-truncating variants (PTVs). The following class of variants in autosomal genes were deemed as “severe”: deletions (partial, whole gene, multigenic), intragenic duplications, nonsense, frameshift, and splice-site variants (4). Since oligogenicity is known to occur in IHH, oligogenic variants were defined as SNVs/indels/CNVs in >1 IHH gene occurring in the same individual of which at least one of the variants was a “severe variant” (see definition above). Secondary oligogenic variants included both “severe” variants and rare missense variants (MAF <1% for variants in autosomal and X-linked recessive genes and MAF <0.1% for variants in autosomal dominant genes).

### Analyses

Categorical (binary) variables are described using percentages, with χ^2^ tests used to compare rates across groups (i.e., fertile eunuch, absent puberty, partial puberty). We used F-tests to identify group differences (at the *p* = 0.01 level). We conducted *post-hoc* tests to compare subgroups, and used the Bonferroni correction to account for the dependency among variables. Quantitative variables are described using descriptive statistics (mean, standard deviation, median, range). Assay results falling below the level of detection were assigned the level of detection for analyses (e.g., LH/FSH = 1.6 IU/L, inhibin B = 15.6 pg/mL). Student’s t-tests and Mann-Whitney U tests were employed to compare hormone levels between groups. For rare variant association testing, the total number of alternate and reference alleles across the coding regions of CHH genes were aggregated into a single alternate allele count (AAC) and reference allele count (RAC) per group. The AACs and RACs were then used in a single rare variant burden test between groups using Fisher exact test. *P* values <0.05 were considered statistically significant.

## Results

A total of 240 men with HH who underwent detailed neuroendocrine phenotyping (i.e., blood sampling Q 10 min. X 12-24 hrs.) were included for analyses. The absent puberty group (TV <4mL) included 139 men while the partial puberty group (TV ≥4mL) had 63 men. A total of 38 men were categorized in the fertile eunuch (FE) group – representing the largest cohort reported to date (**Supplemental Material 1**). The clinical characteristics of the three groups are reported in **Table 1**.

**Table 1:** Comparison of clinical and biochemical characteristics across subsets of men with hypogonadotropic hypogonadism (n=240).

	absent puberty(TV <4mL) n=139	partial puberty(TV ≥4mL) n=63	fertile eunuchn=38
*Clinical characteristics*			
cryptorchidism, n (%)	54 (39%)	18 (29%)	2 (5%) ^A, B^
micropenis, n (%)	40 (29%)	11 (17%)	0 (0%) ^A, B^
anosmia, n (%)	84 (60%)	30 (48%)	16 (42%)
TV (mL), mean±SD	2.2±0.7	6.0±2.3	11.5±2.7 ^A, B^
*Biochemical profile*			
undetectable FSH, n (%)	104 (75%)	29 (46%)	0 (0%) ^A, B^
FSH (IU/L), mean±SD	1.9±1.1	2.7±2.2	3.6±1.8 ^A, C^
undetectable LH, n (%)	117 (84%)	30 (48%)	2 (5%) ^A, B^
LH (IU/L), mean±SD	1.8±0.6	2.6±1.7	4.1±2.1 ^A, B^
apulsatile LH, n (%)	130 (94%)	43 (68%)	8 (21%) ^A, B^
LH amplitude (IU/L), mean±SD	1.6±0.9	1.7±1.1	2.1±1.2
testosterone (ng/dL), mean±SD	31±28	38±32	65±45 ^A, D^
Inhibin B (pg/mL), mean±SD	16±13	15±10	124±72 ^A, B^

TV, testicular volume; FSH, follicle stimulating hormone; LH, luteinizing hormone; A, p<0.0001 vs. absent puberty; B, p<0.0001 vs. partial puberty, C, p<0.05 vs. partial puberty, D, p<0.001 vs. partial puberty.

### Clinical characteristics

All three groups exhibited similar rates of anosmia (i.e., Kallmann syndrome). While there was overlap in TV between the FE and partial puberty groups (range: 8-17.5 vs. 4-15 mL, respectively), the three groups differed significantly according to baseline TV (*p*<0.0001). No men in the FE group had a history of micropenis and only 2/38 (5%) had a history of cryptorchidism – significantly lower than rates observed in both the absent and partial puberty groups (*p*<0.0001). Notably, men with a complete absence of spontaneous puberty (TV <4mL) exhibited the highest rates of cryptorchidism (54/139, 39%) and micropenis (40/139, 29%) – consistent with both absent neonatal activation of the HPG axis (i.e., minipuberty) and absent pubertal HPG axis activation.

### Biochemical profile

When examining biochemical profiles, groups differed in serum testosterone levels – yet all subjects exhibited frankly hypogonadal T levels at presentation (**Table 1**). In relation to serum gonadotropins, the FE group was more likely to have detectable FSH and exhibited significantly higher mean levels of FSH compared to absent and partial puberty groups (both *p*<0.0001). Similarly, of those men with available inhibin B levels, the FE group had significantly higher serum inhibin B levels compared to other groups (124±72 pg/mL, *p*<0.0001). Among the FE men with available inhibin B levels, 12/21 (48%) exhibited serum inhibin B levels well within the normal range. This observation is consistent with the finding of sperm in all samples from FE men who were able to produce an ejaculate. In terms of LH, the FE group was significantly more likely to have detectable LH and higher serum LH levels compared to the other groups (*p*<0.0001). We used dynamic neuroendocrine studies to examine GnRH-induced LH pulsatility, revealing further differences between groups. In total, 30/38 (79%) of men in the FE group exhibited pulsatile LH secretion. In contrast, less than one-third (20/63, 32%) of partial puberty group and very few (9/139, 6%) men with absent puberty exhibited a pulsatile LH secretion pattern. However, when comparing men within each group who exhibited pulsatile LH secretion, neither the median number of pulses (i.e., 3-4 in 12 hours) nor mean pulse amplitude (**Table 1**) differed. Cumulatively, the observations confirm that that the FE group exhibits the mildest neuroendocrine defect of the three groups.

### Fertility outcomes

Of the 240 men with HH, longitudinal data were available on a subset of 77 men who underwent long-term (i.e., ≥12 months) pulsatile GnRH therapy - as previously described (3). In total, 56/77 (73%) developed sperm in the ejaculate. All 11 FE men developed sperm in the ejaculate yet 16/47 (34%) men with absent puberty and 5/19 (26%) with partial puberty remained azoospermic. The maximal sperm count for the FE men (range: 0.5-81 million/mL, median: 11.6 million/mL) was significantly greater than with absent puberty and partial puberty respectively (absent: range 0-289 million/mL, median 2.0 million/mL, *p*=0.006, partial: range 0-49 million/mL, median 1.0 million/mL, *p*=0.002). This retrospective study did not systematically assess the number of men who were attempting to conceive over the 40-year span. Thus, we are only able to report data on those men who actually conceived. In total, 28 men with absent puberty, 22 men with partial puberty, and 11 FE men conceived. All FE men had natural conception without the aid of assisted reproductive technology.

### Reversal of HH

**Figure 1:**
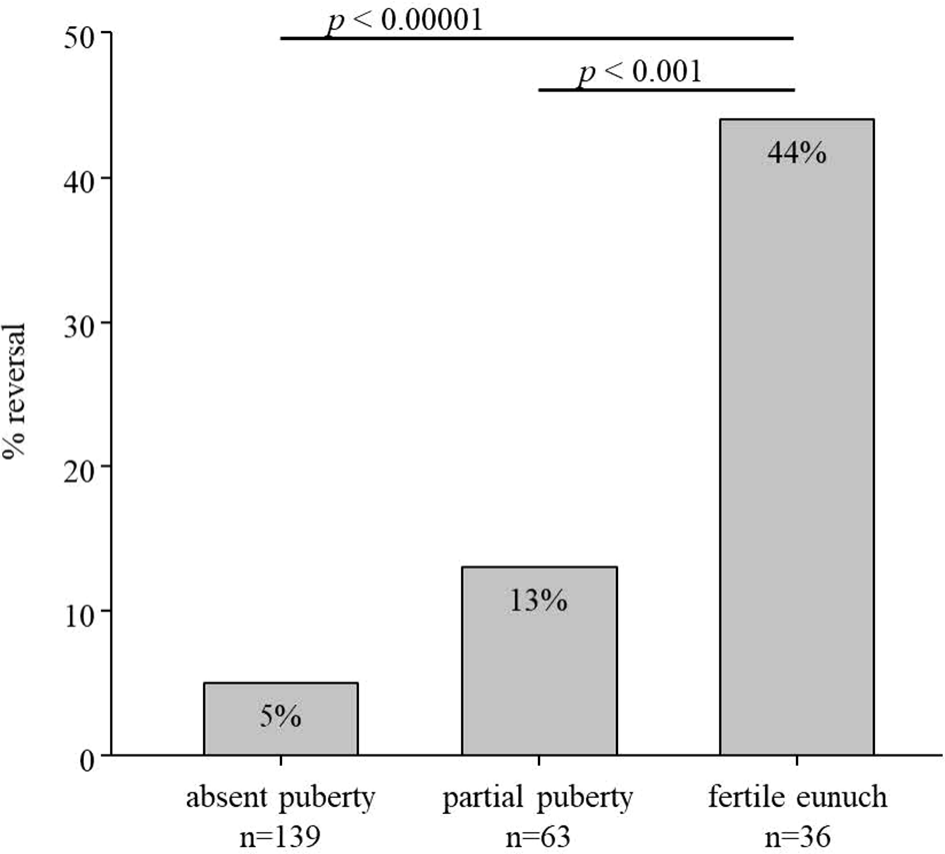
Rate of reversal in subsets of men with hypogonadotropic hypogonadism (n=238). In total, 7/139 men with absent puberty (TV <4mL), 8/63 men with partial puberty (TV >4mL), and 16/36 men with the fertile eunuch syndrome underwent reversal of hypogonadotropic hypogonadism.

Some of the subjects in this HH cohort underwent a treatment washout following either sex steroid replacement or gonadotropin/pulsatile GnRH therapy. Comparing rates of reversal across the three groups revealed striking differences (**Figure 1**). Rates of reversal reported in the literature range between 10-15% of cases (7). Among men with a complete absence of spontaneous puberty, only 7/139 (5%) exhibited reversal; 8/63 (13%) of men with partial puberty underwent reversal. However, in contrast to these two groups, 16/36 (44%) men in the FE group underwent spontaneous reversal of HH (*p*<0.0001) (**Table 2**). Thus, men with the FE sub-set of HH exhibit the mildest reproductive phenotype (i.e., larger TV, higher gonadotropins, pulsatile LH secretion) and significantly higher rates of recovering HPG axis function with sustained testosterone levels in the normal range. When considering all reversals in the cohort (**Table 3**), the age at documented reversal ranged from 19-53 years (median: 23 yrs., mean: 26.3±7.6 yrs.). All men who underwent reversal had been on treatment to normalize serum T levels prior to reversal (treatment duration range: 0.5-19 yrs., median: 4.0 yrs., mean: 5.3±5.0 yrs.). As men were not systematically re-assessed for reversal (i.e. regular treatment washout and hormonal evaluation), we did not make between group comparisons. However, qualitatively, it is interesting to note that reversal typically was documented in early adulthood. Indeed, 22/31 (71%) had reversal noted between the ages of 19 and 29 years (**Table 2**).

**Table 2:** Characteristics of men who underwent reversal of HH (n=31).

	absent puberty(TV <4mL) n=139	partial puberty (TV ≥4mL) n=63	fertile eunuchn=36
*Reversal of HH* n (%)	7 (5%)	8 (13%)	13 (44%)*
*Age at documented reversal*			
range (yrs.)	20-38	19-37	20-53
median (yrs.)	32.0	23.0	23.0
mean±SD (yrs.)	29.6±7.4	24.4±6.5	25.8±8.2
*Treatment duration to documented reversal*			
range (yrs.)	3-19	0.5-17	1-13
median (yrs.)	9.0	3.0	3.0
mean±SD (yrs.)	9.9±6.0	4.5±5.6	3.7±3.0

HH, hypogonadotropic hypogonadism; TV, testicular volume, *p<0.001.

**Table 3:** Summary of genetic findings in subsets of men with hypogonadotropic hypogonadism (n=201).

variants	absent puberty(TV<4mL) n=115	partial puberty(TV≥4mL) n=56	fertile eunuchn=30
*Variants in HH loci (n, %)*			
missense & PTVs	49 (43%)	21 (38%)	13 (43%)
PTVs	24 (21%)	6 (11%)	6 (20%)
*Oligogenic variants (n, %)*			
Oligogenic (PTVs & missense)	31 (31%)	12 (21%)	9(30%)
Oligogenic with at least 1 PTV	13 (11%)	4 (7%)	5 (17%)
*Individual loci (n, %)*			
* ANOS1* PTVs	15 (13%)*	1 (2%)	0 (0%)
* FGFR1* PTVs	4 (3%)	0 (0%)**	3 (10%)
* GNRHR (homozygous)*	2 (2%) †	1 (2%)	3 (10%)

TV, testicular volume; HH, hypogonadotropic hypogonadism; PTV, protein truncating variant,

*p<0.001 vs. fertile eunuch, **p<0.005 vs. fertile eunuch, † p<0.05 vs. fertile eunuch

### Genetic findings and genotype-reproductive phenotype correlations

A subset of 201/240 (84%) men had exome sequencing (ES) data available. We analyzed the exome sequencing data to identify single nucleotide variants (SNVs)/indels and copy number variants (CNVs) in 62 HH-implicated genes (**Supplemental Materials Table 2**). A complete listing of genetic findings of the HH cohort are provided in **Supplemental Materials Table 3**. Protein truncating variants (PTVs) were identified in 36/201 (18%) of subjects (**Table 3**). The majority of PTVs (22/36, 61%) occurred along with secondary (oligogenic) variants. We then correlated PTV burden across groups (i.e., absent puberty, partial puberty, FE). Notably, neither rates of PTVs in the 62 IHH genes nor oligogenicity differed across groups (**Table 3**). Comparing single gene differences across groups revealed three significant findings. First, the absent puberty group was enriched for PTVs in *ANOS1* – and no *ANOS1* variants were found in the FE group. Second, PTVs in *FGFR1* had a bi-modal distribution – appearing in 3% of severe GnRH deficiency cases (i.e., absent puberty) and 10% of the mildest GnRH deficiency (i.e., FE group). Only 1/56 (2%) of subjects in the partial puberty groups harbored a PTV in *ANOS1* (the patient exhibited a TV of 4mL and a quantitative smell testing score of 5% - just above the <5% threshold for anosmia). Third, 10% of men in the FE group harbored homozygous variants in *GNRHR*. Although variants in *FGFR1* and *GNRHR* were not exclusive to the FE subset of HH. **Supplemental Table 4** provides details on the identified *FGFR1* and *GNRHR* PTVs including zygosity, minor allele frequency, *in silico* prediction, and *in vitro* functionality (as well as diagnosis - i.e., nHH/KS). However, of the 13 FE men who underwent reversal, 4/13 (31%) carried variants in *FGFR1* (two PTVs), and 1/13 (8%) harbored a homozygous missense *GNRHR* variant (**Table 3**). **Supplemental Table 5** provides details on reversal cases harboring PTVs in *FGFR1* and *GNRHR* PTVs. **Table 4** presents a summary of genetic findings in subsets of men who underwent reversal of HH. These observations are consistent with prior reports demonstrating that PTVs in *ANOS1* produce a severe reproductive phenotype (i.e., absent puberty) (3, 13). In contrast, variants in *FGFR1* and *GNRHR* may lead to milder reproductive phenotypes including reversible HH.

**Table 4:** Summary of genetic findings in subsets of men with reversal of HH (n=27).

variants	Reversal with absent puberty (TV<4mL) n=6	Reversal with partial puberty (TV≥4mL) n=8	Reversal fertile eunuch n=13
*Variants in HH loci (n, %)*			
missense & PTVs	0 (0%)	2 (25%)	5 (38%)
PTVs	0 (0%)	0 (0%)	2 (15%)
*Oligogenic variants (n, %)*			
Oligogenic (PTVs & missense)	0 (0%)	1 (13%)	4 (31%)
Oligogenic with at least 1 PTV	0 (0%)	0 (0%)	2 (15%)
*Individual loci (n, %)*			
* FGFR1* *	0 (0%)	0 (0%)	4 (31%)
* TACR3* (missense)	0 (0%)	1 (13%)	0 (0%)
* GNRHR* (missense)	0 (0%)	0 (0%)	1 (8%)

TV, testicular volume; PTV, protein truncating variant, * includes PTVs (n=2) and missense (n=2).

## Discussion

Herein we report on the natural history of the largest cohort of men with the FE variant of HH (n=38). The FE subset appears to represent the mildest end of the clinical spectrum of HH. The FE men exhibited significantly larger TV compared to HH men with absent or partial spontaneous puberty. Moreover, rates of cryptorchidism in the FE group were similar to the 3% incidence of cryptorchidism recognized in male infants in the general population. The low rates of cryptorchidism and absence of micropenis suggest the HPG axis was activated during minipuberty – again attesting that FE variant represents the mildest HH phenotype. The preserved mini-puberty in FE cohort is particularly relevant as preserved early life activation of the HPG axis is associated with favorable adult fertility and semen quality (19, 20). Not surprisingly, and as suggested by the term “fertile eunuch”, FE men appear to have the best outcomes for fertility-inducing treatment as cryptorchidism, prepubertal TV (<4ml), and low serum inhibin B are all negative predictors of outcome for fertility in men with HH (21).

Notably, the olfactory phenotype is not a useful characteristic to define the FE subset as rates of anosmia were similar across all three groups. This finding suggests that olfactory phenotypes do not necessarily track with the severity of pubertal phenotypes in HH men. However, we observed striking biochemical differences in the reproductive hormonal milieu across the three groups. The FE group exhibited significantly higher serum LH levels and was more likely to exhibit a pulsatile LH secretion pattern. The significantly higher serum FSH and normal inhibin B levels observed in the FE subset are linked to seminiferous tubule development – as clinically evidenced by larger TV in the FE group. Human studies demonstrate that unopposed FSH administered to HH men induces increased inhibin B levels, testicular growth, and histologic evidence of Sertoli cell and spermatogonia proliferation (22, 23). Interventional studies examining the hormonal dynamics of the initial 7-days of pulsatile GnRH administration to HH men showed an inverse relationship between FSH responsiveness to GnRH and baseline TV and inhibin B levels. Such observations imply that seminiferous tubule maturity constrains FSH responsiveness to GnRH (24).

In early puberty, FSH stimulates Sertoli cells and spermatogonia proliferation which continues until intratesticular T levels trigger the developmental switch in these cells from proliferation to maturation. In the FE men, endogenous GnRH secretion appears to be enfeebled yet is sufficient enough to stimulate gonadotropin release. It is likely that the higher circulating FSH levels in the FE group stimulate progressive testicular seminiferous tubule development – as evidenced by 48% of the FE group exhibiting normal serum inhibin B levels. However, LH stimulus is neither sufficient to induce normal peripheral testosterone levels (i.e., lack of secondary sexual characteristics) nor high intratesticular testosterone levels - enabling continued Sertoli cell and spermatogonia proliferation. The preserved Sertoli cell function and spermatogenesis in the FE cohort challenges the dogma that normal testosterone levels are absolutely essential for spermatogenesis. Indeed, in keeping with these human observations, recent murine studies have elegantly demonstrated completely T-independent spermatogenesis (25). Taken together, undervirilization, a relatively larger TV, hypogonadal T levels, detectable gonadotropins, relatively preserved inhibin-B levels and intact spermatogenesis represent the clinical and biochemical hallmarks of the FE subset.

Piecing together the natural history study data regarding preserved spermatogenesis in the FE cohort and prior experimental data in humans and mice provides indirect support for the rationale behind sequential gonadotropin therapy for fertility induction in men with severe HH (i.e., TV<4mL) (6). A sequential approach to fertility induction in men with severe GnRH deficiency (i.e., TV<4mL) involves pre-treatment with unopposed FSH prior to the addition of either human chorionic gonadotropin (i.e., FSH + hCG) (26, 27) or pulsatile GnRH (22). In essence, the unopposed FSH pre-treatment recapitulates the gonadotropin milieu of early puberty – when nocturnally-entrained GnRH pulses favor FSH secretion spurring testicular growth (i.e., Sertoli cell and spermatogonial proliferation) (28). Several single-center studies suggest that such a sequential approach may improve fertility outcomes in men with severe GnRH deficiency (22, 26, 27, 29, 30) – yet larger, multicenter studies are needed to definitively prove superiority to traditional combined gonadotropin therapy (i.e., hCG + FSH without pre-treatment).

In relation to the genetic findings in the FE cohort, no rare variants in *ANOS1* were identified in the FE group - suggesting that the absence of an *ANOS1* variant is a qualitative negative predictor of the FE sub-type. The absence of *ANOS1* variant in the FE group is perhaps not surprising as prior work has shown that *ANOS1* variants are associated with more severe GnRH deficiency and poorer outcomes for fertility-inducing treatment (13). In contrast, the FE group was enriched for homozygous/compound heterozygous variants in the recessive *GNRHR* gene. Similarly, the FE group was enriched for PTVs in *FGFR1* compared to the partial puberty group. Moreover, of the FE men (with available ES data) who underwent a spontaneous reversal, 4/13 (31%) carry variants in *FGFR1* (two PTVs), and one (8%) harbored a homozygous missense *GNRHR* variant. Thus, variants in *FGFR1* and *GNRHR* appear to confer milder reproductive phenotypes - including reversible HH. These findings highlight the plasticity of the HPG axis. Prior murine studies have shown that constitutively active *fshr* enables androgen-independent spermatogenesis (25). This observation raises the hypothesis that similar genetic variants may underlie the preserved spermatogenesis seen in the FE cohort. However, we neither identified variants in FSH-beta (*FSHB*) nor FSH receptor (*FSHR*). In addition, there are three reports of FE cases harboring a rare variant in the LH receptor (*LHR*) (31–33) – yet we did not identify any such variants in our FE cohort. These findings suggest the preserved spermatogenesis in the FE cohort must result from other cryptic regulatory mechanisms that remain to be deciphered.

The reversible HH phenomenon is characterized by a recovery of HPG axis function and may occur even in the setting of pathogenic variants in HH genes (7). The majority of reported cases of reversal are from single-center case studies (**Supplemental Material Table 6**). Two larger series (15, 34) indicate that reversal occurs in 10-15% of men with HH– yet predictors of reversal have remained elusive. Our finding that 16/36 (44%) of FE men underwent reversal reveals the subtype is enriched for reversible HH. These data are the first empirical evidence of a clinical predictor of reversible HH. As reversal affects only a small subset of the rare patients with HH, further work is needed to validate the findings in an international cohort of reversal cases. Clinically, the present observations bear relevance for the management of men with the FE subtype. These individuals should be monitored for increased TV while on testosterone therapy and/or periodic supervised washouts to assess reversal (1, 2). However, caution is merited as there is evidence that some men who undergo reversal may relapse into a hypogonadal state (34).

Relative strengths of this natural history investigation include the size of the FE cohort - the largest reported to date. Additional strengths include the systematic, detailed clinical characterization phenotyping including cryptorchidism/micropenis, olfactory, pubertal, and neuroendocrine phenotyping as well as the comprehensive screening for variants in HH loci. This study is the first to present a clinical predictor of reversible HH. As with all retrospective studies, there are limitations. the study included patients seen at the Massachusetts General Hospital Reproductive Endocrine Unit (1980-2020). During this time, gonadotropin assay methods shifted. We utilized values gleaned from historical records and anchored results using standards (methods). Further, as this was a single-center study, we cannot exclude the risk of referral bias. While the overall sample size across cohorts is robust for a rare condition such as HH, caution is warranted in extrapolating results as findings may not be representative of all patients with HH.

## Contributions

This is the largest study to date reporting the clinical, biochemical, and genetic characteristics of men with the fertile eunuch (FE) sub-set of hypogonadotropic hypogonadism (HH). Charting the natural history identifies significant phenotypic, hormonal, and genetic signatures that differentiate the FE sub-set from other forms of HH including absent puberty and partial spontaneous puberty. The FE subset represents a mild form of GnRH deficiency in the spectrum of HH. Notably, 16/36 (44%) men in the FE group underwent a spontaneous reversal of HH. As such, this manuscript identifies the FE phenotype as the first identified clinical predictor for reversible HH in adulthood.

## Data availability statement

The original contributions presented in the study are included in the article/**Supplementary Materials**. Further inquiries can be directed to the corresponding author.

## Ethics statement

The studies involving human participants were reviewed and approved by Massachusetts General Hospital Institutional Review Board (MGH-IRB). The patients/participants provided their written informed consent to participate in this study.

## Author contributions

Conceptualization, AD, SS, & RB; methodology, AD, MS, & RB; formal analysis, AD, EA, MS, & LP; validation, SS & RB; investigation, AD, IM, & LP; resources, SS; data curation, AD, MS, IM, & LP writing - original draft preparation, AD; writing - review and editing, MS, IM, EA, LP, SS, & RB; visualization, AD; supervision, AD, SS, & RB; project administration, AD & MS; funding acquisition, AD & SS. All authors have read and agreed to the published version of the manuscript.

## Funding

This work was supported by the National Institutes of Health National Center for Advancing Translational Sciences (1R03TR003533-01) and the National Institutes of Health Eunice Kennedy Shriver National Institute of Child Health and Human Development (1P50HD104224-01) “Massachusetts General hospital – Harvard Center for Reproductive Medicine”.

## Acknowledgments

The authors wish to thank the patients who generously participated in this research. We also acknowledge the nurses of the MGH General Clinical Research Center for their skill and expert care in conducting the detailed neuroendocrine studies.

## Conflict of interest

The authors declare that the research was conducted in the absence of any commercial or financial relationships that could be construed as a potential conflict of interest.

## Publisher’s note

All claims expressed in this article are solely those of the authors and do not necessarily represent those of their affiliated organizations, or those of the publisher, the editors and the reviewers. Any product that may be evaluated in this article, or claim that may be made by its manufacturer, is not guaranteed or endorsed by the publisher.
