# Supplementary material for: Reversible hypogonadotropic hypogonadism in men with the fertile eunuch/Pasqualini syndrome: A single-center natural history study
Source: Front Endocrinol (Lausanne). 2022 Nov 2;13:1054447. doi: 10.3389/fendo.2022.1054447 (PMC9666691; doi:10.3389/fendo.2022.1054447)
Supplement: Supplementary file 1 [file Table_1.docx]

Supplementary Material

# Supplementary Table 1. Summary of published literature on fertile eunuch variant/Pasqualini syndrome

| **Year (ref.)** | **N** | **Age (yrs.)** | **Testicular volume** | **Testis Biopsy** | **Treatment response** | | | **Paternity** | **Genetics** |
| --- | --- | --- | --- | --- | --- | --- | --- | --- | --- |
|  |  |  |  |  | **hCG** | **Clomid** | **GnRH** |  |  |
| 1950 (1) | 1 | 24 | "normal" | spermatogenesis | YES | n/a | n/a | n/a | n/a |
| 1953 (2) | 5 | 21-40 | 2.8 X 1.5 & "small normal" | spermatogenesis | YES | n/a | n/a | YES | n/a |
| 1953 (3) | 1 | 28 | L: 4.0 X 2.0 R: 3.75 X 2.5 | spermatogenesis | n/a | n/a | n/a | n/a | n/a |
| 1954 (4) | 1 | 36 | L: 3.0 X 2.0 X 2.0 R: 2.5 X 2.0 X 2.0 | spermatogenesis | YES | n/a | n/a | n/a | n/a |
| 1955 (5) | 3 | 18, 54, 52 | 3.0 X 2.0 / "normal" / 2.0 X 1.5 | spermatogenesis | YES | n/a | n/a | n/a | n/a |
| 1955 (6) | 2 | 24, 19 | L: 4.0 X 2.5 X 2.5 R: 4.0 X 3.0 X 3.0 & 2.0 X 1.0 X 1.0 (bilat) | spermatogenesis | YES | n/a | n/a | n/a | n/a |
| 1957 (7) | 2 | 36, 18 | L: 3.0 X 2.0 X 2.0 R: 2.5 X 2.0 X 2.0 L: 3.0 X 2.0 X 2.0 R: 3.0 X 2.5 X 2.0 | spermatogenesis | n/a | n/a | n/a | n/a | n/a |
| 1961 (8) | 1 | 18 | "both testes measured 4.0 cm" | n/a | YES | n/a | n/a | n/a | n/a |
| 1964 (9) | 1 | 33 | "normal" | spermatogenesis | YES | n/a | n/a | n/a | n/a |
| 1965 (10) | 1 | 25 | 1.0 X 1.5 | spermatogenesis | YES | n/a | n/a | n/a | n/a |
| 1966 (11) | 1 | 27 | 5.0 X 3.0 | spermatogenesis | YES | n/a | n/a | n/a | n/a |
| 1967 (12) | 3 | 20, 28, 20 | 4mL & 8 mL & 16 mL | spermatogenesis | n/a | n/a | n/a | n/a | n/a |
| 1968 (13) | 1 | 24 | 5.5 X 2.0 | spermatogenesis | YES | NO | n/a | n/a | n/a |
| 1971 (14) | 2 | 21, 28 | 3.0 X 3.0 & 2.5 X 3.0 | spermatogenesis | n/a | NO | n/a | n/a | n/a |
| 1972 (15) | 9 | 14-26 | 7mL / 20mL / 15mL / 12mL / 7mL / 6mL / 14mL / 7mL / 4mL | spermatogenesis | YES (a) | n/a | n/a | n/a | n/a |
| 1972 (16) | 1 | 27 | "borderline normal" | spermatogenesis | YES | n/a | n/a | n/a | n/a |
| 1974 (17) | 2 | 34, 26 | "normal" | spermatogenesis | YES | n/a | YES (b) | n/a | n/a |
| 1974 (18) | 1 | 36 | 10mL | spermatogenesis | YES | n/a | n/a | n/a | n/a |
| 1975 (19) | 1 | 23 | 5.2 & 4.4 mL | spermatogenesis | YES | NO | n/a | n/a | n/a |
| 1975 (20) | 1 | 31 | 4.0 X 2.0 | spermatogenesis | YES | n/a | YES | n/a | n/a |
| 1976 (21) | 2 | 28, 31 | 2.5 X 3.0 / 3.0 X 4.0 | spermatogenesis | n/a | n/a | n/a | n/a | n/a |
| 1977 (22) | 2 | 21, 23 | "normal" (photos for each) | spermatogenesis | YES | NO | YES | n/a | n/a |
| 1978 (23) | 3 | 20, 27, 36 | 4.0 X 3.5 / 3.5 X 2.5 / L: 3.6 X 2.4 & R: 3.6 X 2.2 | spermatogenesis | YES | n/a | n/a | n/a | n/a |
| 1980 (24) | 2 | 28, 27 | 3.5 X 2.0 & 3.0 X 2.0 / “small” | spermatogenesis | YES | n/a | n/a | YES | n/a |
| 1994 (25) | 1 | 23 | 6 mL | n/a | YES | n/a | n/a | n/a | n/a |
| 1996 (26) | 1 | 19 | "~1 cm in length (NL: 4-5 cm)" | n/a | YES | n/a | YES | YES | n/a |
| 2001 (27) | 1 | 26 | 17 mL | n/a | YES | n/a | n/a | n/a | *GNRHR* |
| 2002 (28) | 6* | n/a | ">12mL" | n/a | n/a | n/a | n/a | n/a | n/a |
| 2003 (29) | 1 | 19 | 18mL | spermatogenesis | YES | n/a | n/a | YES | *LHB* |
| 2004 (30) | 1 | 30 | 8mL | spermatogenesis | YES | n/a | YES | YES | *LHB* |
| 2007 (31) | 1 | 67 | 5.0 X 5.0 | n/a | n/a | n/a | n/a | n/a | n/a |
| 2009 (32) | 1 | 43 | "normal" | spermatogenesis | n/a | n/a | n/a | n/a | *LHB* |

n: number fertile eunuch/Pasqualini syndrome patients, hCG: human chorionic gonadotropin; GnRH: gonadotropin-releasing hormone; *GNRHR*: GnRH receptor variant; *LHB*: LB beta variant; (a): six patients responded to hCG, three were treated with testosterone; (b) patients responded to luteinizing-releasing hormone (LH-RH); * fertile eunuch patients were reported (without details) in a study reporting on a HH cohort (KS: n=30, nHH: n=42, AHH: n=6).

**References**

1. Pasqualini RQ, Bur, G. E. Síndrome hipoandrogénico con gametogénesis conservada. Revista de la Asociacion Medica Argentina. 1950;64:6-10.

2. McCullagh EP, Beck JC, Schaffenburg CA. A syndrome of eunuchoidism with spermatogenesis, normal urinary FSH and low or normal ICSH: (fertile eunuchs). J Clin Endocrinol Metab. 1953;13(5):489-509.

3. Landau RL. Hypogonadism with spermatogenesis; a case report. J Clin Endocrinol Metab. 1953;13(5):510-8.

4. Rosemblit ED. Insufficiencia androgenica con espermatogenesis conservada. La Prensa Medica Argentina. 1954;41:1825-29.

5. Albert A, Underdahl, L. O., Greene, L. F., Lorenz, N. Male hypogonadism VII: The testis in partial gonadotropic failure during puberty (lack of luteinizing hormone only). Proceedings of the Staff Meetings of the Mayo Clinic. 1955;30(2):31-43.

6. Pasqualini RQ, Bur G. Hypoandrogenic syndrome with spermatogenesis. Fertil Steril. 1955;6(2):144-57.

7. Rosemblit E, Hojman, D. Sindrome de eunucoidisimo con espermatogenesis conservada. La Semana Medica. 1957(December):1295-303.

8. Witte JJ, Schwarz F. Fertile eunuchoidism caused by an arrest of pubertal development. Acta Endocrinol (Copenh). 1961;36:462-6.

9. Biliczki F, Svarzas, F., David, M. A., Kovacs, K. . Ein kranheitsfal vonfertilem eunuchoidism. Medizinische Klinik. 1964;59(August):1346-53.

10. Davies AG. Eunuchoid treated with gonadotrophins. Proceedings of the Royal Society of Medicine. 1965;58(8):18.

11. Meyhofer W. Prepubertal ICSH deficiency [Der praepuberale ICSH mangel]. Arch Klinishe Experimentelle Dermatologie. 1966;227(1):678-84.

12. Johnsen SG. The mechanisms invovled in testicular degeneration in man. Acta Endocrinologica. 1967:17-40.

13. Faiman C, Hoffman DL, Ryan RJ, Albert A. The "fertile eunuch" syndrome: demonstration of isolated luteinizing hormone deficiency by radioimmunoassay technique. Mayo Clin Proc. 1968;43(9):661-7.

14. Santen RJ, Leonard JM, Sherins RJ, Gandy HM, Paulsen CA. Short- and long-term effects of clomiphene citrate on the pituitary-testicular axis. J Clin Endocrinol Metab. 1971;33(6):970-9.

15. Christiansen P. Urinary gonadotrophins in nine fertile eunuchs. Acta Endocrinol (Copenh). 1972;71(3):454-68.

16. Kjessler B. Repeated paternity subsequent to distinct courses of hcg and testosterone in a hypoandrogenic male with preserved spermatogenesis, a "fertile eunuch". Andrologie. 1972;4(3):219-29.

17. Bonati B, Marrama, P., Della Casa, P., editor "The "fertile eunuch" syndrome: Primary of hypogonadotrophic dissociated hypogonadism? The Endocrine Function of the Human Testes; 1974; Florence, Italy. London, UK: Academic Press Inc; 1974.

18. Hornstein OP, Becker H, Hofmann N, Kleissl HP. [Pasqualini-syndrome ("fertile eunuchoidism"). Clinical, histological and hormone-analytical findings]. Dtsch Med Wochenschr. 1974;99(39):1907-11 passim.

19. del Pozo E, Bolte E, Very M. Suprasellar disturbance in the syndrome of fertile eunuchoidism: case report. Acta Endocrinol (Copenh). 1975;80(1):165-70.

20. Williams C, Wieland RG, Zorn EM, Hallberg MC. Effect of synthetic gonadotropin-releasing hormone (GnRH) in a patient with the "fertile eunuch" syndrome. J Clin Endocrinol Metab. 1975;41(1):176-9.

21. Boyar RM, Wu RH, Kapen S, Hellman L, Weitzman ED, Finkelstein JW. Clinical and laboratory heterogeneity in idiopathic hypogonadotropic hypogonadism. J Clin Endocrinol Metab. 1976;43(6):1268-75.

22. Makler A, Glezerman M, Lunenfeld B. The fertile eunuch syndrome. An isolated leydig-cell failure? Andrologia. 1977;9(2):163-70.

23. Smals AG, Kloppenborg PW, van Haelst UJ, Lequin R, Benraad TJ. Fertile eunuch syndrome versus classic hypogonadotrophic hypogonadism. Acta Endocrinol (Copenh). 1978;87(2):389-99.

24. Rogol AD, Mittal KK, White BJ, McGinniss MH, Lieblich JM, Rosen SW. HLA-compatible paternity in two "fertile eunuchs" with congenital hypogonadotropic hypogonadism and anosmia (the Kallmann syndrome). J Clin Endocrinol Metab. 1980;51(2):275-9.

25. Kung AW, Zhong YY, Lam KS, Wang C. Induction of spermatogenesis with gonadotrophins in Chinese men with hypogonadotrophic hypogonadism. Int J Androl. 1994;17(5):241-7.

26. Wortsman J, Hughes LF. Case report: olfactory function in a fertile eunuch with Kallmann syndrome. Am J Med Sci. 1996;311(3):135-8.

27. Pitteloud N, Boepple PA, DeCruz S, Valkenburgh SB, Crowley WF, Jr., Hayes FJ. The fertile eunuch variant of idiopathic hypogonadotropic hypogonadism: spontaneous reversal associated with a homozygous mutation in the gonadotropin-releasing hormone receptor. J Clin Endocrinol Metab. 2001;86(6):2470-5.

28. Pitteloud N, Hayes FJ, Boepple PA, DeCruz S, Seminara SB, MacLaughlin DT, et al. The role of prior pubertal development, biochemical markers of testicular maturation, and genetics in elucidating the phenotypic heterogeneity of idiopathic hypogonadotropic hypogonadism. J Clin Endocrinol Metab. 2002;87(1):152-60.

29. Shiraishi K, Naito K. Fertile eunuch syndrome with the mutations (Trp8Arg and Ile15Thr) in the beta subunit of luteinizing hormone. Endocr J. 2003;50(6):733-7.

30. Valdes-Socin H, Salvi R, Daly AF, Gaillard RC, Quatresooz P, Tebeu PM, et al. Hypogonadism in a patient with a mutation in the luteinizing hormone beta-subunit gene. N Engl J Med. 2004;351(25):2619-25.

31. Zugor V, Dimmler A, Schrott KM, Schott GE. [Pasqualini's syndrome]. Aktuelle Urol. 2007;38(4):320-3.

32. Achard C, Courtillot C, Lahuna O, Meduri G, Soufir JC, Liere P, et al. Normal spermatogenesis in a man with mutant luteinizing hormone. N Engl J Med. 2009;361(19):1856-63.

# Supplementary Table 2. List of 62 HH genes

| Gene | Ensembl Transcript | RefSeq | Mode of Inheritance |
| --- | --- | --- | --- |
| *AMH* | ENST00000221496.4 | NM_000479.5 | Autosomal recessive |
| *AMHR2* | ENST00000257863.4 | NM_020547.3 | Autosomal dominant |
| *ANOS1* | ENST00000262648.3 | NM_000216.4 | X-linked recessive |
| *AXL* | ENST00000301178.4 | NM_021913.5 | Autosomal dominant |
| *CCDC141* | ENST00000420890.2 | NM_173648 | Autosomal recessive |
| *CHD7* | ENST00000423902.2 | NM_017780.4 | Autosomal dominant |
| *CUL4B* | ENST00000404115.3 | NM_001079872.2 | X-linked recessive |
| *DCC* | ENST00000442544.2 | NM_005215.4 | Autosomal dominant |
| *DMXL2* | ENST00000543779.2 | NM_001378457.1 | Autosomal recessive |
| *DUSP6* | ENST00000279488.7 | NM_001946.4 | Autosomal dominant |
| *FEZF1* | ENST00000442488.2 | NM_001024613.4 | Autosomal recessive |
| *FGF17* | ENST00000359441.3 | NM_003867.4 | Autosomal dominant |
| *FGF8* | ENST00000320185.2 | NM_033163.5 | Autosomal dominant |
| *FGFR1* | ENST00000447712.2 | NM_023110 | Autosomal dominant |
| *FLRT3* | ENST00000378053.3 | NM_198391.3 | Autosomal dominant |
| *GLCE* | ENST00000261858.2 | NM_015554.3 | Autosomal dominant |
| *GLI3* | ENST00000395925.3 | NM_000168.6 | Autosomal dominant |
| *GNRH1* | ENST00000276414.4 | NM_001083111.2 | Autosomal recessive |
| *GNRHR* | ENST00000226413.4 | NM_000406.3 | Autosomal recessive |
| *HS6ST1* | ENST00000259241.6 | NM_004807.3 | Autosomal dominant |
| *IGSF10* | ENST00000282466.3 | NM_178822.5 | Autosomal dominant |
| *IL17RD* | ENST00000296318.7 | NM_017563.5 | Autosomal recessive |
| *KISS1* | ENST00000367194.4 | NM_002256.4 | Autosomal recessive |
| *KISS1R* | ENST00000234371.5 | NM_032551.5 | Autosomal recessive |
| *KLB* | ENST00000257408.4 | NM_175737.4 | Autosomal dominant |
| *LEP* | ENST00000308868.4 | NM_000230.3 | Autosomal recessive |
| *LEPR* | ENST00000349533.6 | NM_002303.6 | Autosomal recessive |
| *NDNF* | ENST00000379692.4 | NM_024574.4 | Autosomal dominant |
| *NR0B1* | ENST00000378970.4 | NM_000475.5 | X-linked recessive |
| *NRP1* | ENST00000265371.4 | NM_003873.7 | Autosomal dominant |
| *NRP2* | ENST00000360409.3 | NM_003872.3 | Autosomal dominant |
| *NSMF* | ENST00000371475.3 | NM_001130969.3 | Autosomal recessive |
| *NTN1* | ENST00000173229.2 | NM_004822.3 | Autosomal dominant |
| *OTUD4* | ENST00000454497.2 | NM_001102653.1 | Autosomal recessive |
| *PCSK1* | ENST00000311106.3 | NM_000439.5 | Autosomal recessive |
| *PLXNA1* | ENST00000393409.2 | NM_032242.4 | Autosomal dominant |
| *PNPLA6* | ENST00000414982.3 | NM_001166111.1 | Autosomal recessive |
| *POLR3A* | ENST00000228347.4 | NM_018082.6 | Autosomal recessive |
| *POLR3B* | ENST00000228347.4 | NM_018082.6 | Autosomal recessive |
| *PROK2* | ENST00000295619.3 | NM_001126128.2 | Autosomal recessive |
| *PROKR2* | ENST00000217270.3 | NM_144773 | Autosomal recessive |
| *PROP1* | ENST00000308304.2 | NM_006261.5 | Autosomal recessive |
| *RAB18* | ENST00000356940.6 | NM_021252.5 | Autosomal recessive |
| *RAB3GAP1* | ENST00000442034.1 | NM_012233.3 | Autosomal recessive |
| *RAB3GAP2* | ENST00000358951.2 | NM_012414.4 | Autosomal recessive |
| *RMST* | ENST00000541282.1 | NR_024037 | Autosomal dominant |
| *RNF216* | ENST00000389902.3 | NM_207111.4 | Autosomal recessive |
| *SEMA3A* | ENST00000265362.4 | NM_006080.3 | Autosomal dominant |
| *SEMA3E* | ENST00000643230.1 | NM_012431 | Autosomal dominant |
| *SEMA7A* | ENST00000261918.4 | NM_003612.5 | Autosomal dominant |
| *SMCHD1* | ENST00000320876.6 | NM_015295.3 | Autosomal dominant |
| *SOX10* | ENST00000396884.2 | NM_006941.4 | Autosomal dominant |
| *SOX2* | ENST00000325404.1 | NM_003106.4 | Autosomal dominant |
| *SPRY4* | ENST00000344120.4 | NM_001127496.3 | Autosomal dominant |
| *SRA1* | ENST00000336283.6 | NM_001035235.4 | Autosomal recessive |
| *STUB1* | ENST00000219548.4 | NM_005861.4 | Autosomal recessive |
| *TAC3* | ENST00000458521.2 | NM_013251.4 | Autosomal recessive |
| *TACR3* | ENST00000304883.2 | NM_001059.3 | Autosomal recessive |
| *TBC1D20* | ENST00000354200.4 | NM_144628.4 | Autosomal recessive |
| *TCF12* | ENST00000438423.2 | NM_207037.2 | Autosomal dominant |
| *TUBB3* | ENST00000315491.12 | NM_006086.4 | Autosomal dominant |
| *WDR11* | ENST00000263461.6 | NM_018117.12 | Autosomal dominant |

# Supplemental Table 3. List of variants identified in genetic sequencing

| Group: Fertile Eunuch variant |
| --- |
| *NSMF* c.587G>A p.R196H het; *FGFR1* c.1864C>T p.R622* het; |
| *CHD7* c.8228_8247dup p.F2750Lfs*4 het; |
| *FGFR1* c.296A>G p.Y99C het; |
| *FGFR1* c.1037_1038del p.S346Yfs*61 het; DCC c.619A>G p.I207V het; |
| *GNRHR* c.317A>G p.Q106R hom; *LEPR* c.390G>C p.Q130H het; *DMXL2* c.4187C>T p.S1396F het; |
| *GNRHR* c.416G>A p.R139H het; *GNRHR* c.95C>T p.T32I het; |
| *CHD7* c.3366G>C p.K1122N het; *POLR3A* c.3337A>G p.I1113V het; |
| *GNRHR* c.317A>G p.Q106R het; *GLI3* c.1161del p.P388Qfs*13 het; *CCDC141* c.4369G>A p.V1457I het; |
| *FGFR1* c.1916T>C p.I639T het; *CCDC141* c.182A>G p.K61R het; |
| *FGFR1* c.1279G>T p.V427L het; *RAB3GAP2* c.590G>A p.R197Q het; |
| *FGFR1* c.1809C>A p.C603* het; *LEPR* c.2246T>C p.L749S het; |
| *TBC1D20* c.1016T>C p.M339T het; P*OLR3B* c.1244T>C p.M415T het; *POLR3B* c.2818-2A>T het; *KL* c.560C>T p.P187L het; |
| *GNRHR* c.317A>G p.Q106R het; *GNRHR* c.286C>T p.P96S het; |
| Group: Partial Puberty |
| *FGFR1* c.1936C>G p.R646G het; |
| *IL17RD* c.56A>G p.N19S het; *ARHGAP35* c.860A>G p.N287S het; *TCF12* c.454C>T p.P152S het; *SMCHD1* c.35C>A p.A12D het; |
| *SOX10* c.1038_1039delAC p.P347Tfs*54 het; |
| *GNRHR* c.636C>G p.N212K het; *GNRHR* c.317A>G p.Q106R het; *PROP1* c.425C>T p.A142V het; |
| *PROP1* c.425C>T p.A142V het; *KLB* c.926G>A p.R309Q het; |
| *SOX10* c.713C>T p.P238L het; *GNRHR* c.436C>T p.P146S het; *NDNF* c.40C>A p.P14T het; *IGSF10* c.1786C>T p.H596Y het; |
| *POLR3A* c.3013C>T p.R1005C het; *POLR3A* c.2830G>T p.E944* het; |
| *SOX10* c.1038_1039delAC p.P347Tfs*54 het; *KLB* c.3032T>C p.L1011P het; |
| *CHD7* c.7952A>G p.N2651S het; |
| *TCF12* c.1491dup p.V498Cfs*12 het; *CCDC141* c.3111A>T p.K1037N het; |
| *PROKR2* c.779C>T p.T260M het; *PROKR2* c.332T>G p.M111R het; |
| *SMCHD1* c.1186C>A p.Q396K het; |
| *CHD7* c.6956G>A p.R2319H het; |
| *ANOS1* c.1369C>T p.R457* hem; *CUL4A* c.86T>C p.V29A het; *PNPLA6* c.1123G>C p.A375P het; *AMH* c.136C>G p.P46A het; |
| *FGFR1* c.2011G>C p.A671P het; |
| *ANOS1* c.1759G>T p.V587L hem; *TCF12* c.391T>C p.S131P het; |
| *FGFR1* c.1447C>A p.P483T het; *NDNF* c.785del p.S262* het; *CCDC14*1 c.1521A>C p.Q507H het; *DMXL2* c.812C>G p.T271S het; |
| *AMH* c.211C>T p.R71W hom; |
| *PROKR2* c.518T>G p.L173R het; *FGFR1* c.374C>T p.S125L het; *OTUD4* c.2689C>T p.H897Y het; |
| *FGFR1* c.2172C>G p.N724K het; *FGFR1* c.2165C>A p.P722H het; *SQSTM1* c.1088G>A p.G363E het; *POLR3B* c.2369T>G p.V790G het; *SMCHD1* c.3106G>A p.V1036I het; |
| *TACR3* c.766T>C p.Y256H hom; *IGSF10* c.4631C>T p.S1544F hom; |
| Group: Absent Puberty |
| *FGFR1* c.2107G>C p.G703R het; *POLR3A* c.3734G>A p.R1245Q het; |
| *ANOS1* c.1201_1207+4del11 p.N401Kfs*5 hem; |
| *CHD7* c.2185A>G p.K729E het; *RNF216* c.1717C>T p.R573C het; |
| *ANOS1* c.571C>T p.R191* hem; *RAB3GAP1* c.1233_1235del p.L412del het; |
| *FGFR1* c.1285-2A>G het; *WDR11* c.1175G>A p.R392Q het; |
| *KISS1R* c.991C>T p.R331* het; *KISS1R* c.1195T>A p.*399R het; *SQSTM1* c.374A>G p.N125S het; *PNPLA6* c.1198G>A p.D400N het; |
| *AMH* c.1213G>C p.A405P het; *CHD7* c.6287A>G p.H2096R het; *DMXL2* c.7179_7181del p.V2394del het; |
| *FGFR1* c.304G>A p.V102I het; *KL* c.499C>T p.R167C het; |
| *CHD7* c.8366C>T p.A2789V het; *FGFR1* c.1854G>T p.K618N het; *GNRHR* c.785G>A p.R262Q het; |
| *CHD7* c.1175C>T p.S392F het; |
| *CHD7* c.3160C>T p.R1054W het; |
| *ANOS1* c.67_92del p.L23Cfs*77 hem; *GNRH1* c.229C>T p.R77* het; *DCC* c.2267G>A p.R756Q het; *CCDC141* c.1802A>T p.H601L het; |
| *CHD7* c.8405G>A p.G2802E het; *CHD7* c.7861C>G p.Q2621E het; *TYRO3* c.1025G>A p.W342* het; |
| *SEMA3A* c.708C>G p.D236E het; *PCSK1* c.2257A>C p.N753H het; *DMXL2* c.6067C>G p.P2023A het; |
| *GNRHR* c.416G>A p.R139H het; *GNRHR* c.95C>T p.T32I het; |
| *ANOS1* c.1801del p.L601Yfs*19 hem; *SMCHD1* c.1804A>G p.I602V het; |
| *FGFR1* c.1016A>G p.Y339C het; *IGSF10* c.7853C>T p.T2618M het; *SEMA7A* c.1863C>G p.F621L het; |
| *ANOS1* c.1449+1G>A hem; *GLI3* c.1285C>G p.P429A het; |
| *FGFR1* deletion, AXL c.5C>T p.A2V het; *AMH* c.295A>T p.T99S het; |
| *FGFR1* c.2038C>T p.Q680* het; |
| *ANOS1* c.1887_1888del p.Y630Pfs*36 hem; *PLXNA1* c.1765G>A p.V589M het; *PNPLA6* c.1484C>T p.P495L het; |
| *HS6ST1* c.968G>A p.R323Q het; *SMCHD1* c.1049A>T p.Y350F het; |
| *FGFR1* c.1025T>C p.L342S het; |
| *FGFR1* c.710G>A p.G237D het; *PNPLA6* c.4108G>A p.G1370S het; *CHD7* c.5533G>A p.G1845R het; |
| *PROK2* c.163del p.I55* hom; *OTUD4* c.386C>G p.A129G het; |
| *ANOS1* deletion, *PROP1* c.152G>T p.G51V het; |
| *IL17RD* c.2009C>T p.P670L het; *RNF216* c.2251C>T p.R751C het; *RNF216* c.2149C>T p.R717C het; |
| *PNPLA6* c.2389G>A p.V797M het; *SMCHD1* c.1571A>G p.N524S het; |
| *NR0B1* c.1253C>T p.T418M hem; *NDNF* c.901G>A p.V301I het; *KLB* c.2959G>A p.V987I het; |
| *CHD7* c.2095A>G p.S699G het; |
| *FGFR1* c.1097C>T p.P366L het; *KLB* c.820A>G p.I274V het; |
| *ANOS1* c.1449+1G>A hem; *CHD7* c.1117C>T p.L373F het; |
| *ANOS1* deletion, SRA1 c.266C>T p.P89L het; *PNPLA6* c.1484C>T p.P495L het; |
| *ANOS1* c.814C>T p.R272* hem; *DCC* c.3470A>G p.H1157R het; |
| *FGFR1* c.2241C>A p.F747L het; *FGFR1* c.2302G>C p.D768H het; *PLXNA1* c.2159G>A p.G720E het; |
| *ANOS1* c.1381delC p.R461Gfs*21 hem; *FGF8* c.159_164del p.T54_V55del het; *AXL* c.2570C>T p.A857V het; *AMH* c.1666G>A p.E556K het; |
| *ANOS1* c.1270C>T p.R424* hem; *RAB3GAP1* c.2938G>A p.A980T het; *DMXL2* c.6046G>A p.D2016N het; |
| *SPRY4* c.530A>G p.K177R het; *CHD7* c.7957C>T p.R2653* het; *RAB3GAP2* c.585A>G p.I195M het; |
| *RAB3GAP2* c.4060A>G p.I1354V het; *LEPR* c.371A>G p.D124G het; *CHD7* c.6190A>G p.I2064V het; |
| *FGFR1* c.764C>T p.P255L het; *AXL* c.2119C>T p.R707C het; |
| *ANOS1* c.1891C>T p.R631* hem; |
| *FGFR1* c.1727_1734del p.R576Pfs*77 het; *OTUD4* c.1458G>T p.K486N het; *OTUD4* c.909C>A p.S303R het; |
| *POLR3B* c.497-2A>G het; *POLR3B* c.1244T>C p.M415T het; |
| *FGFR1* c.2057T>C p.F686S het; *SQSTM1* c.712A>G p.K238E het; |
| *ANOS1* c.1A>C p.? hem; |
| *GNRHR* c.112C>T p.R38* hom; |
| *SEMA3A* c.1922A>T p.Q641L het; *RAB3GAP1* c.1006C>T p.R336C het; *CCDC141* c.4036C>T p.R1346W het; |
| *ANOS1* c.1392_1405del p.E465Qfs*30 hem; |
| *ANOS1* c.1551C>G p.F517L hem; |

**Supplemental Table 4:** Characteristics of *GNRHR* and *FGFR1* variants identified in the absent puberty, partial puberty, and fertile eunuch groups.

| **Diagnosis** | **Gene** | **Variant** | **Zygosity** | **Minor allele frequency** | ***in silico* predictions**  **Polyphen-2 SIFT CADD** | | | ***in vitro* functional studies** |
| --- | --- | --- | --- | --- | --- | --- | --- | --- |
| Group: Absent puberty | | | | | | | | |
| nHH | *GnRHR* | c.416G>A, p.R139H | heterozygous | 0.0001 | probably damaging | deleterious | 32 | Yes (1) |
|  |  | c.95C>T, p.T32I | heterozygous | novel | probably damaging | deleterious | 25.2 | Yes (2) |
| nHH | *GnRHR* | c.112C>T, p.R38* | homozygous | 0.0002 | N/A | N/A | 37 | N/A |
| KS | *FGFR1* | *c.1727_1734del*  *p.R576Pfs*77* | heterozygous |  | N/A | N/A |  | N/A |
| nIHH | *FGFR1* | *c.1285-2A>G* | heterozygous | novel | N/A | N/A | 33 | N/A |
| KS | *FGFR1* | copy number variation | N/A | N/A | N/A | N/A | N/A | N/A |
| nIHH | *FGFR1* | *c.2038C>T p.Q680** | heterozygous | novel | N/A | N/A | 43 | N/A |
| Group: Partial Puberty | | | | | | | | |
| nHH | *GnRHR* | c.636C>G p.N212K | heterozygous | novel | probably damaging | deleterious | 23.7 | N/A |
|  |  | c.317A>G p.Q106R | heterozygous | 0.005 | probably damaging | deleterious | 24.3 | Yes (3) |
| Group: Fertile Eunuch | | | | | | | | |
| nHH | *GnRHR* | c.317A>G p.Q106R | homozygous | 0.005 | probably damaging | deleterious | 24.3 | Yes (3) |
| KS | *GnRHR* | c.416G>A p.R139H | heterozygous | 0.0001 | probably damaging | deleterious | 32 | Yes (1) |
|  |  | c.95C>T p.T32I | heterozygous | novel | probably damaging | deleterious | 25.2 | Yes(2) |
| nHH | *GnRHR* | c.317A>G p.Q106R  c.286C>T p.P96S | heterozygous  heterozygous | 0.005  0.0001 | probably damaging  probably damaging | deleterious  deleterious | 24.3  25.4 | Yes (3)  N/A |
| KS | *FGFR1* | c.1864C>T p.R622* | heterozygous | novel | N/A | N/A | 39 | N/A |
| KS | *FGFR1* | c.1037_1038del  p.S346Yfs*61 | heterozygous | 4.00E-06 | N/A | N/A | NA | N/A |
| nIHH | *FGFR1* | c.1809C>A p.C603* | heterozygous | novel | N/A | N/A | 34 | N/A |

nHH: normosmic hypogonadotropic hypogonadism; KS: Kallmann syndrome

**References**

1. Leanos-Miranda, A., et al., In vitro coexpression and pharmacological rescue of mutant gonadotropin-releasing hormone receptors causing hypogonadotropic hypogonadism in humans expressing compound heterozygous alleles. *J Clin Endocrinol Metab*, 2005. 90(5): p. 3001-8.

2. de Roux, N., et al., A family with hypogonadotropic hypogonadism and mutations in the gonadotropin-releasing hormone receptor. *N Engl J Med*, 1997. 337(22): p. 1597-602.

3. Costa, E.M., et al., Two novel mutations in the gonadotropin-releasing hormone receptor gene in Brazilian patients with hypogonadotropic hypogonadism and normal olfaction. *J Clin Endocrinol Metab*, 2001. 86(6): p. 2680-6.

**Supplemental Table 5:** Characteristics of the *GNRHR* and *FGFR1* variants in the group FE men with reversal (n=5).

| **Diagnosis** | **Gene** | **Variant** | **Zygosity** | **Minor allele frequency** | ***in silico* predictions**  **Polyphen-2 SIFT CADD** | | | ***in vitro* functional studies** |
| --- | --- | --- | --- | --- | --- | --- | --- | --- |
| KS | *FGFR1* | c.1864C>T p.R622* | heterozygous | novel | N/A | N/A | 39 | N/A |
| nHH | *FGFR1* | c.296A>G p.Y99C het | heterozygous | novel | deleterious | probably damaging | 28.9 | N/A |
| nHH | *FGFR1* | c.1279G>T p.V427L het | heterozygous | novel | tolerated | benign | 27.4 | N/A |
| nHH | *FGFR1* | c.1809C>A p.C603* het | heterozygous | novel | N/A | N/A | 34 | N/A |
| nHH | *GNRHR* | c.317A>G p.Q106R hom | homozygous | 0.005 | probably damaging | deleterious | 24.3 | Yes (1) |

nHH: normosmic hypogonadotropic hypogonadism; KS: Kallmann syndrome

**References**

1. Costa, E.M., et al., Two novel mutations in the gonadotropin-releasing hormone receptor gene in Brazilian patients with hypogonadotropic hypogonadism and normal olfaction. *J Clin Endocrinol Metab*, 2001. 86(6): p. 2680-6.

**Supplemental Table 6. Published reports of reversible hypogonadotropic hypogonadism**

| **Year (ref.)** | **Report type** | **Number of reversals** | **Genetic findings**  **(of 62 genes in Supplemental Table 2)** |  |
| --- | --- | --- | --- | --- |
| 1975 (1) | Case report | 1 | n/a |  |
| 1980 (2) | Case series | 2 | n/a |  |
| 1983 (3) | Case report | 1 | n/a |  |
| 1986 (4) | Case report | 1 | n/a |  |
| 1994 (5) | Case report | 1 | n/a |  |
| 1996 (6) | Case report | 1 | n/a |  |
| 1996 (7) | Case report | 1 | n/a |  |
| 1999 (8) | Cohort | 5 | n/a |  |
| 2001 (9) | Case report | 1 | *GNRHR* |  |
| 2002 (10) | Case report | 1 | *GNRHR* |  |
| 2005 (11) | Case report | 1 | *FGFR1* |  |
| 2007 (12) | Case report | 1 | *ANOS1* |  |
| 2007 (13) | Cohort | 15 | *FGFR1,* *GNRHR* |  |
| 2008 (14) | Case report | 1 | *PROKR2* |  |
| 2010 (15) | Cohort | 10 | *TAC3, TACR3* |  |
| 2011 (16) | Case report | 1 | *HS6ST1* |  |
| 2012 (17) | Cohort | 6 | *CHD7, FGFR1,* *GNRHR* |  |
| 2013 (18) | Case report | 1 | *GNRHR* |  |
| 2013 (19) | Cohort | 9 | n/a |  |
| 2013 (20) | Cohort | 2 | *IL17RD, SPRY4* |  |
| 2014 (21) | Cohort | 18 | n/a |  |
| 2014 (22) | Cohort | 44 | *FGFR1, GNRHR, PROKR2, TACR3, TAC3, HS6ST1, NELF* |  |
| 2014 (23) | Case series | 2 | n/a |  |
| 2014 (24) | Cohort | 1 | *GNRHR* |  |
| 2016 (25) | Case report | 1 | *SOX10* |  |
| 2017 (26) | Case report | 1 | n/a |  |
| 2017 (27) | Case report | 1 | *PROK2* |  |
| 2019 (28) | cohort | 6 | *GNRHR, IGSF1* |  |
| 2020 (29) | Cohort | 1 | *CCDC141* |  |
| 2022 (30) | Cohort | 13 | *CHD7, IL17RD, PROKR2* |  |

**References**

1. Rezvani I, DiGeorge AM, Rutano J, Snyder PJ. Delayed puberty and anosmia: Coincidence or Kallmann variant? [abstract]. *Ped Res*. 1975;9:244.

2. Rogol AD, Mittal KK, White BJ, McGinniss MH, Lieblich JM, Rosen SW. HLA-compatible paternity in two "fertile eunuchs" with congenital hypogonadotropic hypogonadism and anosmia (the Kallmann syndrome). *J Clin Endocrinol Metab.* 1980;51(2):275-9.

3. Rowe RC, Schroeder ML, Faiman C. Testosterone-induced fertility in a patient with previously untreated Kallmann's syndrome. *Fertil Steril*. 1983;40(3):400-1.

4. Bauman A. Markedly delayed puberty or Kallmann's syndrome variant. *Andrology*. 1986;7(4):224-7.

5. Bagatell CJ, Paulsen CA, Bremner WJ. Preservation of fertility despite subnormal gonadotropin and testosterone levels after cessation of pulsatile gonadotropin-releasing hormone therapy in a man with Kallmann's syndrome. *Fertil Steril*. 1994;61(2):392-4.

6. Wortsman J, Hughes LF. Case report: olfactory function in a fertile eunuch with Kallmann syndrome*. Am J Med Sci*. 1996;311(3):135-8.

7. Kadva A, Di WL, Djahanbakhch O, Monson J, Silman R. Evidence for the Bauman variant in Kallmann's syndrome. *Clin Endocrinol (Oxf).* 1996;44(1):103-10.

8. Quinton R, Cheow HK, Tymms DJ, Bouloux PM, Wu FC, Jacobs HS. Kallmann's syndrome: is it always for life? *Clin Endocrinol (Oxf).* 1999;50(4):481-5.

9. Pitteloud N, Boepple PA, DeCruz S, Valkenburgh SB, Crowley WF, Jr., Hayes FJ. The fertile eunuch variant of idiopathic hypogonadotropic hypogonadism: spontaneous reversal associated with a homozygous mutation in the gonadotropin-releasing hormone receptor. *J Clin Endocrinol Metab*. 2001;86(6):2470-5.

10. Dewailly D, Boucher A, Decanter C, Lagarde JP, Counis R, Kottler ML. Spontaneous pregnancy in a patient who was homozygous for the Q106R mutation in the gonadotropin-releasing hormone receptor gene. *Fertil Steril*. 2002;77(6):1288-91.

11. Pitteloud N, Acierno JS, Jr., Meysing AU, Dwyer AA, Hayes FJ, Crowley WF, Jr. Reversible kallmann syndrome, delayed puberty, and isolated anosmia occurring in a single family with a mutation in the fibroblast growth factor receptor 1 gene. *J Clin Endocrinol Metab*. 2005;90(3):1317-22.

12. Ribeiro RS, Vieira TC, Abucham J. Reversible Kallmann syndrome: report of the first case with a KAL1 mutation and literature review. *Eur J Endocrinol.* 2007;156(3):285-90.

13. Raivio T, Falardeau J, Dwyer A, Quinton R, Hayes FJ, Hughes VA, et al. Reversal of idiopathic hypogonadotropic hypogonadism. *N Engl J Med*. 2007;357(9):863-73.

14. Sinisi AA, Asci R, Bellastella G, Maione L, Esposito D, Elefante A, et al. Homozygous mutation in the prokineticin-receptor2 gene (Val274Asp) presenting as reversible Kallmann syndrome and persistent oligozoospermia: case report. *Hum Reprod.* 2008;23(10):2380-4.

15. Gianetti E, Tusset C, Noel SD, Au MG, Dwyer AA, Hughes VA, et al. TAC3/TACR3 mutations reveal preferential activation of gonadotropin-releasing hormone release by neurokinin B in neonatal life followed by reversal in adulthood. *J Clin Endocrinol Metab*. 2010;95(6):2857-67.

16. Tornberg J, Sykiotis GP, Keefe K, Plummer L, Hoang X, Hall JE, et al. Heparan sulfate 6-O-sulfotransferase 1, a gene involved in extracellular sugar modifications, is mutated in patients with idiopathic hypogonadotrophic hypogonadism. *Proc Nat Acad Sci (USA)*. 2011;108(28):11524-9.

17. Laitinen EM, Tommiska J, Sane T, Vaaralahti K, Toppari J, Raivio T. Reversible congenital hypogonadotropic hypogonadism in patients with CHD7, FGFR1 or GNRHR mutations. *PloS one*. 2012;7(6):e39450.

18. Tommiska J, Jorgensen N, Christiansen P, Juul A, Raivio T. A homozygous R262Q mutation in the gonadotropin-releasing hormone receptor presenting as reversal of hypogonadotropic hypogonadism and late-onset hypogonadism. *Clin Endocrinol (Oxf)..* 2013;78(2):316-7.

19. Kulshreshtha B, Khadgawat R, Gupta N, Ammini A. Progression of puberty after initiation of androgen therapy in patients with idiopathic hypogonadotropic hypogonadism. *Indian J Endocrinol Metab*. 2013;17(5):851-4.

20. Miraoui H, Dwyer AA, Sykiotis GP, Plummer L, Chung W, Feng B, et al. Mutations in FGF17, IL17RD, DUSP6, SPRY4, and FLRT3 Are Identified in Individuals with Congenital Hypogonadotropic Hypogonadism. *Am J Hum Genet*. 2013;92(5):725-43.

21. Mao JF, Xu HL, Duan J, Chen RR, Li L, Li B, et al. Reversal of idiopathic hypogonadotropic hypogonadism: a cohort study in Chinese patients. *Asian J Androl*. 2014.

22. Sidhoum VF, Chan YM, Lippincott MF, Balasubramanian R, Quinton R, Plummer L, et al. Reversal and relapse of hypogonadotropic hypogonadism: resilience and fragility of the reproductive neuroendocrine system. *J Clin Endocrinol Metab*. 2014;99(3):861-70.

23. Santhakumar A, Balasubramanian R, Miller M, Quinton R. Reversal of isolated hypogonadotropic hypogonadism: long-term integrity of hypothalamo-pituitary-testicular axis in two men is dependent on intermittent androgen exposure. *Clin Endocrinol (Oxf).*. 2014;81(3):473-6.

24. Tommiska J, Kansakoski J, Christiansen P, Jorgensen N, Lawaetz JG, Juul A, et al. Genetics of congenital hypogonadotropic hypogonadism in Denmark. *Eur J Med Genet*. 2014;57(7):345-8.

25. Maione L, Brailly-Tabard S, Nevoux J, Bouligand J, Young J. Reversal of congenital hypogonadotropic hypogonadism in a man with Kallmann syndrome due to SOX10 mutation. *Clin Endocrinol (Oxf).* 2016;85(6):988-9.

26. Rashid O, Ram N, Farooq S, Kiran Z. Idiopathic hypogonadotropic hypogonadism reversal after testosterone replacement in a 34-year-old male. *BMJ Case Rep*. 2017;2017.

27. Pierzchlewska MM, Robaczyk MG, Vogel I. Induction of puberty with human chorionic gonadotropin (hCG) followed by reversal of hypogonadotropic hypogonadism in Kallmann syndrome. *Endokrynol Pol*. 2017;68(6):692-6.

28. Amato LGL, Montenegro LR, Lerario AM, Jorge AAL, Guerra Junior G, Schnoll C, et al. New genetic findings in a large cohort of congenital hypogonadotropic hypogonadism. *Eur J Endocrinol*. 2019;181(2):103-19.

29. Hou Q, Wu J, Zhao Y, Wang X, Jiang F, Chen DN, et al. Genotypic and phenotypic spectrum of CCDC141 variants in a Chinese cohort with congenital hypogonadotropic hypogonadism. *Eur J Endocrinol*. 2020;183(3):245-54.

30. Sun T, Xu W, Chen Y, Niu Y, Wang T, Wang S, et al. Reversal of idiopathic hypogonadotropic hypogonadism in a Chinese male cohort. *Andrologia*. 2022:e14583.
